# End group chemistry modulates physical properties and biomolecule release from biodegradable polyesters

**DOI:** 10.1039/d5tb00816f

**Published:** 2025-08-06

**Authors:** Matthew A. Borrelli, Jordan J. P. Warunek, Tarini Ravikumar, Stephen C. Balmert, Steven R. Little

**Affiliations:** a Department of Chemical and Petroleum Engineering, University of Pittsburgh PA 15261 USA; b McGowan Institute for Regenerative Medicine, University of Pittsburgh PA 15261 USA; c Tomas E. Starzl Transplantation Institute, University of Pittsburgh School of Medicine PA 15261 USA; d Department of Surgery, University of Pittsburgh School of Medicine PA 15261 USA; e Department of Immunology, University of Pittsburgh School of Medicine PA 15261 USA; f Department of Bioengineering, University of Pittsburgh PA 15261 USA; g Department of Dermatology, University of Pittsburgh School of Medicine, Pittsburgh PA 15213 USA; h Department of Pharmaceutical Sciences, University of Pittsburgh PA 15261 USA; i Department of Ophthalmology, University of Pittsburgh PA 15261 USA; j Clinical and Translational Science Institute, University of Pittsburgh PA 15261 USA

## Abstract

Long-acting injectable protein therapeutics are a rapidly advancing arm of pharmaceuticals. A promising and versatile class of such formulations involves encapsulation of therapeutic protein within poly(lactic-*co*-glycolic acid) (PLGA) degradable microparticles (MP) to shield the protein from enzymatic degradation and control the release rate. However, models based on degradation and erosion of PLGA polymer matrices do not always fully capture release behavior, due in part to electrostatic interactions between the polymer terminal group and encapsulated compound. The repertoire of functionalized PLGA polymers commercially available has now expanded to include terminal group chemistries that may significantly alter polymer characteristics including charge, hydrophobicity, and erosion. This work aims to explore how PLGA terminal group chemistry affects polymer physical properties and charged biomolecule release kinetics. PLGA with hydroxyl (PLGA–OH), amine (PLGA–NH_2_), or carboxylic acid (PLGA–COOH) terminal groups that have neutral, positive, or negative charge, respectively, were evaluated. Experiments assessing the physical properties of the polymers indicate PLGA–NH_2_ has reduced hydrophobicity, degrades faster, exhibits emulsion stabilizing behavior, and has reduced phagocytic clearance by bone marrow derived macrophages. Charged biomolecule release rates are increased from PLGA–NH_2_ MPs and slightly accelerated from PLGA–OH MPs, compared to PLGA–COOH MPs. These studies provide further insight into the interactions between charged biomolecules and the encapsulating polymer and could provide additional tools to tune release for various protein therapeutics that experience such interactions.

## Abstract 2

Poly(lactic-*co*-glycolic acid) terminated with charged terminal groups physical property assessment, quantification of formed microparticle degradation and uptake by phagocytes, and measurement of biomolecule release.

## Graphical abstract

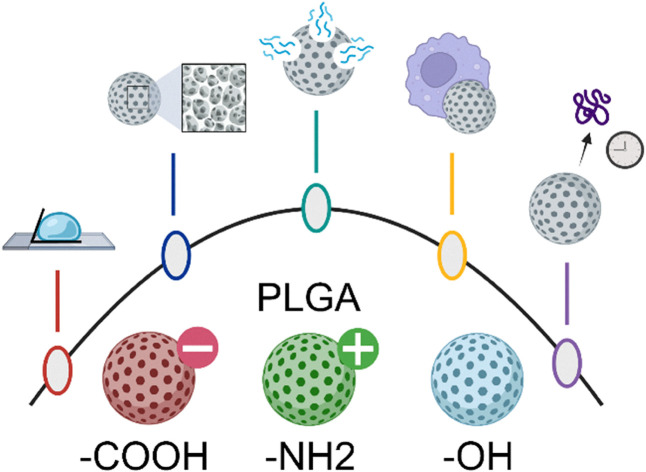

## Introduction

The market for protein-based therapeutics was $265 billion in 2021 and is expected to expand to $394.2 billion by 2026 [BCC Research Manual Citation].^1^ Many of these protein drugs are limited in their delivery and application due to limited oral bioavailability and half-lives often lasting minutes.^2^ Consequently, when delivered through the parenteral route, frequent injections, are required to facilitate these treatments, leading to compliance issues.^3^ In recent years, significant effort have been made to develop long-acting injectable formulations of protein drugs to help reduce the need for frequent dosing.^4^ One strategy to achieve long-term release is to encapsulate protein and peptide therapeutics within polymer microspheres that can serve as a depot at a local site of injection.

One of the most widely explored degradable polymers for delivery of protein therapeutics is poly(lactic-*co*-glycolic acid) (PLGA), which has an excellent track record of safety in FDA-approved formulations.^5^ Encapsulation of proteins within PLGA microparticles (MP) can significantly improve bioavailability while providing controlled release through tunable degradation kinetics.^6–8^ Indeed PLGA and other polymers have been gaining increasing interest as platforms for treatment in disease.^4,9–14^ The release kinetics of molecules encapsulated within PLGA MP are largely erosion controlled, where the polymer matrix presents a physical barrier that gradually erodes and becomes more porous as the polymer degrades.^15^ Early models characterizing the rate of PLGA degradation have focused on the breakdown of the polymer matrix^16^ and have been validated with degradation studies.^17^

However, accounting for degradation and erosion of the polymer matrix alone cannot always accurately predict biomolecule release, due in part to interactions between the polymer and encapsulated compound.^18^ More specifically, proteins and peptides are biomolecules with charge that can interact with the polymer matrix, delaying or halting the release of the active agent.^19–22^ We have previously reported that the encapsulated peptide or protein charge can change throughout the course of PLGA degradation due to a local change in micro environmental pH.^19^ Thus, this is a poorly controlled phenomenon occurring during biomolecule release that requires innovation to address.

In efforts to mitigate protein/peptide and polymer interactions, approaches such as super charging proteins,^23^ protein PEGylation,^24^ and co-encapsulation of acylation inhibitors^21,25^ have been developed. However, these approaches can complicate the development of protein releasing PLGA formulations due to high cost, increased design complexity, and/or reducing protein loading capacity. Thus, mitigating polymer-protein interactions without modifying the protein or reducing potential loading could improve the translatability of microparticle-based delivery of proteins.

We hypothesized that manipulating the PLGA terminal group with a neutral or positive charged group may be used to modulate peptide/protein and polymer interactions. Accordingly, we sought to explore the effect of polymer end-group chemistry on polymer physical properties as well as MP degradation (*i.e.*, intraparticle pH evolution and changes in molecular weight) and release kinetics. In particular, we studied three low molecular weight (∼4–8 kDa) PLGA polymers with high densities of either –COOH, –NH_2_, or –OH end-groups. Our results indicate that PLGA polymer terminal group chemistry not only influences polymer physical properties but also can significantly modulate biomolecule release kinetics. Thus, the work described herein could provide insight into additional tools that can be used to modulate protein/peptide release rates.

## Experimental

### Materials

Poly(d,l-lactic-*co*-glycolic acid) (PLGA) polymers, with 50 : 50 lactide : glycolide composition, 5 kDa molecular weight, and possessing different end groups (COOH, NH_2_, OH) were purchased from Nanosoft Polymers (Winston-Salem, NC). Two peptides – PKCε peptide substrate (Prok-051, positive peptide) and CDK7tide (Prok-056, neutral peptide) fluorescently labeled with 5-carboxytetramethyl-rhodamine (5-TAMRA) were obtained from CPC Scientific (San Jose, CA). Carrier-free recombinant murine CCL22 (mCCL22) was obtained from R&D systems (Minneapolis, MN).

### Measurement of wetted contact angle (WCA)

Glass slides were dip coated with polymer samples solvated in acetone (100 mg mL^−1^). Samples were then dried for 72 hours at 50 °C in a vacuum oven, after which time they were positioned on an optical tensiometer (VCA-Optima; AST Products Inc.). One (1) μL water (de-ionized) droplets were deposited onto the polymer film, an image was captured, and the contact angle of the droplet was measured using the software package included with the VCA-Optima instrument.

### Differential scanning calorimetry (DSC)

Polymer samples weighing 10–20 mg were heated from −20 °C to 150 °C at a rate of 10 °C min^−1^ in DSC 250 (TA Instruments, New Castle, DE) located in the University of Pittsburgh Nanofabrication and Characterization Core Facility. The heat flux was quantified, and the glass transition temperature was determined using Trios software package.

### Fourier-transform infrared spectroscopy

FTIR spectra of end-capped PLGA polymer were obtained using attenuated total reflection Fourier transformed infrared spectroscopy (ATR-FTIR) on the Nicolet^TM^ iS50R Research FTIR Spectrometer (ThermoFisher).

### Proton nuclear magnetic resonance (H-NMR) spectroscopy

H-NMR measurements were performed for the end-capped PLGA polymers using a Bruker Avance III spectrometer (Bruker Scientific, San Jose, CA) at a resonance frequency of 400 MHz. All spectra were acquired at room temperature with PLGA polymers solvated in deuterated dichloromethane (CD_2_CL_2_).

### Microparticle (MP) formulation

Microparticles (MPs) containing fluorescently labeled model peptides (cationic or neutral charge), a cationic therapeutic protein (CCL22), or blanks were formulated using a double emulsion-evaporation technique, as described previously.^26,27^ Briefly, MPs were prepared by mixing 200 μL of an aqueous solution containing the respective agent (125 μg of fluorescently labeled peptide, 5 μg of CCL22) with 200 mg of PLGA–PLGA–COOH, PLGA–OH, or PLGA–NH_2_ – and dissolved in 4 mL of dichloromethane. The resulting mixture of protein and polymer was sonicated (Active Motif EpiShear probe sonicator, 110 V) at 55% amplitude for 10 s to form the first emulsion (water-in-oil, w/o), and then transferred to a 2% PVA solution (60 mL) and homogenized (L4RT-A; Silverson, East Longmeadow, MA) at 3000 rpm for 1 minute, forming the second emulsion (w/o/w). Following homogenization, the solution was transferred to a 1% (w/v) PVA solution (80 mL) and stirred at 600 rpm for 3 hours on ice to allow the DCM to evaporate. Freshly formed MPs were centrifuged (Eppendorf 5810R 15 amp version, 200 × *g* for 5 min at 4 °C) and washed four times (4×) with 4 °C MilliQ water (Milli-Q IQ 7000, Millipore Sigma) (35 mL). The MPs were then re-suspended in MilliQ water (5 mL), flash-frozen with liquid nitrogen (5 minutes), and lyophilized (Benchtop Pro, Virtis SP Scientific) (<100 mTorr, 48–72 hours). These microparticles were formulated with an osmolarity of 15 mOsm in the inner aqueous phase to produce surface pores, referred to as “porous MP”. Select studies, as stated, formulated microparticles without an osmotic gradient, which are referred to as “non-porous MP” in the text.

### Microparticle characterization and release assays

Scanning electron micrographs of MPs were obtained using a scanning electron microscope (ZEISS Sigma500 VP), located in the University of Pittsburgh Nanofabrication and Characterization Core Facility. MP samples were prepared for SEM imaging by pre-treating with a coating of silver for 60 seconds at 20 mAmps (Denton Sputter Coater). Size distributions of MPs were determined using volume impedance measurements on a Beckman Coulter Counter (Multisizer-3; Beckman Coulter; Brea, CA). *In vitro* release behavior for all MP formulations was characterized by incubating 10 mg of MPs in 1 mL of release media (1% (w/v) bovine serum albumin (BSA) in PBS) and incubated on a roto-shaker (Thermo Scientific™ Tube Revolver Rotator, 1.5–2 mL Eppendorf tube paddles, speed 10) at 37 °C. At specified time intervals, MP suspensions were centrifuged (580*g* for 5 min at room temperature) (Eppendorf 5417R), 800 μL of the supernatant was removed and replaced with 800 μL of fresh release media, and the MPs were resuspended and placed back on the roto-shaker. Supernatant concentrations of released agents were quantified by fluorescence spectrophotometry (SpectraMax M5; Molecular Devices; Sunnyvale, CA) for fluorescently labeled peptides or enzyme-linked immunosorbent assay (ELISA; R&D Systems) for CCL22. Release profiles generated from measured concentrations of peptide or protein were normalized to the total amount loaded within MPs (quantified by solvating MPs and extracting the protein/peptide liberated) and MP mass.

### Quantification of primary amines by fluorescamine assay

Fluorescamine, a non-fluorescent dye, reacts with primary amines to yield a fluorescent (400/475 nm; ex/em) reaction product as reported in various applications with PLGA polymers functionalized to contain amines.^28–30^ Briefly, 500 μL of a 9E-04 M solution of fluorescamine solvated in acetone was used to solvate 20 mg of PLGA–NH_2_ MP or PLGA–COOH MP control. PLGA–COOH was selected as the control because it does not have any amine compounds and most closely resembles PLGA–NH_2_ as it degrades. After 5 minutes, the fluorescent intensity (400/475 nm; ex/em) was measured for *N* = 3 samples and normalized to the acetone/fluorescamine solvent.

### Microparticle zeta potential quantification

Microparticle samples were suspended in de-ionized water (1 mg mL^−1^) and the zeta potential was analyzed (Zetasizer nano317, Malvern, UK). Each measurement was repeated in triplicate.

### Polymer molecular weight measurement during microparticle degradation

Blank MP (20 mg) formulated using the various end-cap (COOH, OH, NH_2_) PLGA were suspended in 2 mL of 100 mM HEPES buffer supplemented with 1% BSA and 150 mM NaCl (Buffered to pH 7.4 with NaOH). At the specified time points, samples were centrifuged (5000 × *g*) and the supernatant was removed. The remaining pellets were washed 3 times (3×) with MilliQ water to remove any entrained buffer. The samples were then lyophilized for 48–72 h. The dried samples were solvated in HPLC-grade tetrahydrofuran (THF) at a concentration of 20 mg mL^−1^. Molecular weights and dispersion were obtained on a TOSOH HLC-8320GPC EcoSEC equipped with two columns (TSKgel-G3000H, TSKgel-G3000H). A mobile phase of THF at 50 °C with a flow rate of 1 mL min^−1^ was used, reported molecular weights were obtained with a refractive index detector (TOSOH) and are relative to polystyrene standards (90, 50, 30, 9, 5, 2.5 kDa).

### pH measurements during microparticle degradation

Ten (10) mg of MP were suspended in 100 mM HEPES buffer supplemented with 1% BSA and 150 mM NaCl (buffered to pH 7.4 with NaOH) and incubated, without media replacement, at 37 °C on an end-over-end rotator. Supernatant: at specified timepoints, samples were collected and centrifuged (2320g, 5 min) the supernatant was collected, and the pH was directly analyzed (pH meter: Thermofisher Orion 3 Star; pH probe: Thermo Scientific™ Orion™ ROSS™ PerpHecT™ Micro Glass Bodied Combination pH Electrode). Intraparticle: 800 μL of acetonitrile (ACN) was added to the precipitated MPs and the samples were vortexed for 10 min to dissolve the MPs. Following dissolution, the samples were centrifuged (2320*g*, 5 min) again to remove any undissolved MP and the supernatant was collected and combined with 200 μL DI water. A pH correlation between dissolved lactic acid monomers solvated in 100 mM HEPES and lactic acid monomers solvated in the resultant HEPES/ACN/DI water (representative of dissolved MP) was generated to calculate the intraparticle pH as previously described.^19,31,32^

### Macrophage co-culture with microparticles

Bone marrow obtained from femurs of three wildtype C57BL/6 mice were cultured with 20 ng mL^−1^ macrophage colony stimulating factor (MCSF) (Biolegend) for 7 days at 37 °C and 5% CO_2_, with complete media changes containing MCSF every 48 hours. On day 7, bone marrow-derived macrophages (BMDMΦs) were cultured with complete media alone (M0/untreated), 100 ng mL^−1^ of LPS and 20 ng mL^−1^ IFN-γ (M1 control), 20 ng mL^−1^ of IL-4 (M2 control), or with 0.1 mg mL^−1^ of each end-capped PLGA for 72 hours. In a separate experiment, BMDMΦs were cultured with smaller (<5 μm diameter) microparticles loaded with Alexa Fluor 680 labeled dextran (ThermoFisher) to determine the internalization fraction. BMDMΦs were cultured for 24 hours with 0.1 mg mL^−1^ of each end-capped PLGA or M0, M1, and M2 control culturing conditions described previously.

#### Study approval

All procedures completed at the University of Pittsburgh were approved by the IACUC of the University of Pittsburgh (protocol 22051178) and complied with the NIH's Guide for the Care and Use of Laboratory Animals (National Academies Press, 2011).

### Flow cytometry

BMDMΦs and their culture media, including the microparticles, were harvested and stained in the assay culture plate using PBS containing extracellular antibodies and eBioscience Fixable viability dye. After washing, cells were fixed with 2% PFA for 10 minutes at room temperature. For intracellular staining, cells were permeabilized with 0.5% saponin. Data was acquired using an Aurora (Cytek Biosciences) and analyzed using FlowJo v10.9.0 (BD Life Sciences). Microparticles (CD11b^−^ fraction of events) were gated out and BMDMΦs (Live cells, CD11b^+^ F4/80^+^) were analyzed.

### Flow antibodies

Pacific Blue anti-mouse F4/80 (1 : 200 Biolegend Cat: 123124; Clone: BM8), BUV395 anti-mouse MHCII (IA/IE) (1 : 200 BD OptiBuild Cat: 743876; Clone: 2G9), APC-eFluor780 anti-mouse CD11b (1 : 200 Invitrogen Cat: 47-0112-82; Clone: M1/70), BV650 anti-mouse CD86 (1 : 200 BD Horizon Cat: 564200 Clone: GL1), AF700 IL-12 p40 (1 : 100 Biolegend Cat: 505214; Clone: C15.6), eBioscience^TM^ fixable viability dye eFluor^TM^ 506 (1 : 500 Thermo Fisher Cat: 65-0866-14).

### ImageStream cytometry

BMDMΦs and their culture media, including the fluorescently labeled (Alexa Fluor 680 Dextran) microparticles were isolated and stained with FITC (CD11b), APC-Cy7 (fixable viability dye), and DAPI. Microparticles and cells were acquired on the Cytek Amnis ImageStream Mk II Imaging Flow Cytometer (Cytek Bio). IDEAS version 6.2 software was used to gate the live cell population and then run its internalization calculation on the gated population. Briefly, the software calculates a ratio of the internalizing probe intensity (MP, Alexa Fluor 680) and the cellular membrane stain (CD11b, FITC). A larger ratio indicates greater internalization.

### Biomolecule net charge predictions

Net charge of peptides and proteins (*Z*), which is based on the protonation state of amino acid side groups and the C- and N-termini, was calculated as a function of pH, similar to our prior work.^19^ Briefly the calculation accounts for the C- and N-termini p*K*_a_ and the p*K*_a_ of positively and negatively charged side chains according to the published amino acid residue structure for the peptide or protein in question. Residue structure for the fluorescently labeled peptides is documented by the supplier (CPC Scientific), and CCL22 structure is posted on <uniport.org> (Accession #: Q9QZU2). Calculated charge was normalized to the total molar mass of the peptide or protein. For convenience, the sequences are as follows: Prok-051 (PKCε peptide, positive peptide) sequence: 5TAMRA-ERMRPRKRQGSVRRRV-NH_2_; Prok-056 (CDK7tide, neutral peptide) sequence: 5TAMRA-YSPTSPSYSPTSPSYSPTSPS; CCL22 Sequence: MSNLRVPLLV ALVLLAVAIQ TSDAGPYGAN VEDSICCQDY IRHPLPSRLV KEFFWTSKSC RKPGVVLITV KNRDICADPR QVWVKKLLHK LS.

### Statistical methods

All release assay experiments were performed in triplicate, and data represent means with standard deviation error bars. For multiple comparisons, one-way Anova was performed followed by Šídák's multiple comparisons test. Comparisons between groups over time were made using two-way ANOVA followed by Tukey's multiple comparisons test. Differences in means were considered to be significant if *p* ≤ 0.05.

## Results

### Material characterization of end-capped PLGA

**Fig. 1:**
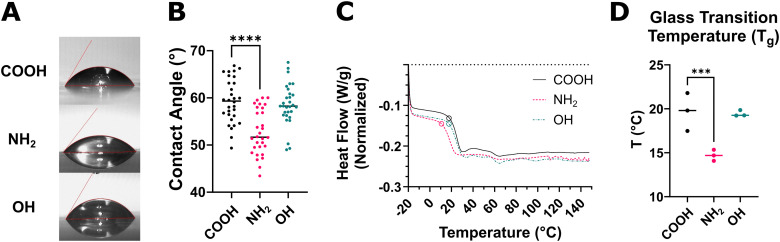
Amine functionalized PLGA displays altered physical properties. PLGA polymers terminated with carboxylic acid (–COOH), amine (–NH_2_), and hydroxyl (–OH) groups were characterized. (A) Representative images and (B) quantitative analysis of water droplet contact angle on polymer films shows PLGA–NH_2_ reduces the surface tension (contact angle) of water (*N* = 30). (C) Representative DSC analysis for −20 to 150 °C at 10 °C min^−1^ ramp for each of the PLGA polymers is shown, in which the circle denotes the glass transition temperature (*T*_g_). (D) The measured *T*_g_ from *N* = 3 independent experiments shows reduced *T*_g_ for PLGA–NH_2_. Statistical comparisons were made using one-way ANOVA followed by Šídák's multiple comparisons test ***p ≤ 0.001, ****p ≤ 0.0001.

Material properties of commercially available 5 kDa molecular weight PLGA polymers terminated with carboxylic acid (–COOH), amine (–NH_2_), and hydroxyl (–OH) groups were assessed. Water contact angle (WCA) measurements were made for water droplets applied to dip coated slides of the polymers. It was observed that water droplets on PLGA–NH_2_ exhibited smaller contact angles indicating reduced hydrophobicity compared to PLGA–COOH (Fig. 1A and B). Further analysis using differential scanning calorimetry (DSC) analysis shows lower glass transition (*T*_g_) temperature for PLGA–NH_2_ compared to the other PLGA (Fig. 1C and D). Additional characterization data includes ATR-FTIR emission spectra for the polymers (Fig. S1A), polymer molecular weight distributions (Fig. S1B), and polymer NMR (Fig. S1C).

### The influence of PLGA end groups on microparticle microstructure

**Fig. 2:**
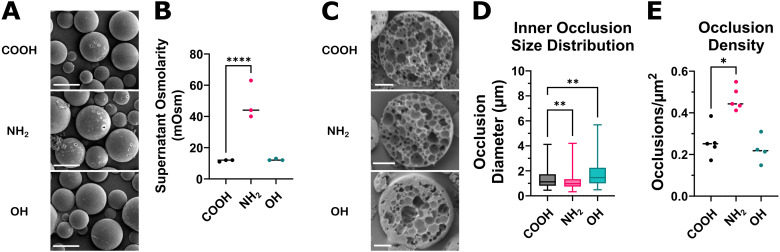
Amine-terminated PLGA exhibits emulsion stabilization and osmotic behavior during microparticle formulation. A series of experiments were conducted to determine how PLGA end-caps impact MP formation during double emulsion formulation procedures. (A) Initial evaluation of microparticles (MP) formulated with deionized water for the inner aqueous phase (non-porousMP) shows increased pore formation with PLGA–NH_2_ microparticles. Scale bar is 10 μm, 1000× magnification. (B) PLGA was incubated in deionized water for 8 h to allow any low *M*_w_ polymer fragments to dissolve. Measured osmolarity of the supernatant shows PLGA–NH_2_ has greater osmolarity than then other PLGA polymers. (C) Representative cross sections of microparticles formulated with an inner aqueous phase osmolarity of 15 mOsm (porousMP). Scale bar is 5 μm, 2000× magnification. (D) Inner occlusion diameter and (E) occlusion density was determined by analyzing the SEM images (C) in QuPath. Inner occlusion diameter is reduced for PLGA–NH_2_ MPs and increased for PLGA–OH MPs, relative to PLGA–COOH MPs, while the density of occlusions within PLGA–NH_2_ microparticles is increased. Results represent the average distribution of inner occlusions for *N* = 4 to 6 microparticle cross sections. Statistical comparisons were made using one-way ANOVA followed by Šídák's multiple comparisons test *p ≤ 0.05, **p ≤ 0.01, ****p ≤ 0.0001.

Double-emulsion (w/o/w) MPs were formulated with the various end-capped PLGA polymers to determine if the terminal group impacts the particle size, surface morphology, and inner microstructure. We first formulated blank microparticles (MP) with ultra-pure, de-ionized water for the inner aqueous phase. Scanning electron microscopy (SEM) imaging of these MP revealed surface pore formation had occurred, sparingly, in PLGA–COOH MP and was prevalent in PLGA–NH_2_ MP (Fig. 2A). Since MP surface porosity can result from an osmotic gradient between the inner and outer aqueous phases (*e.g.*, inner aqueous phase with greater osmolarity),^27^ we sought to determine whether the observed differences in surface porosity could be attributed to differential partitioning of low *M*_w_ PLGA oligomers in the inner aqueous phase. To evaluate this possibility, the different end-cap PLGA polymers were incubated in water for 8 hours, followed by osmolarity determination for the supernatants. Notably, the dissolution of PLGA–NH_2_ oligomers in water was significantly greater than PLGA–COOH or PLGA–OH, as evidenced by elevated osmolarity of the corresponding aqueous supernatant (Fig. 2B). In order to formulate MPs from the different polymers with similar surface porosity, we next formulated MPs using salt to create an inner aqueous phase with an osmolarity of 15 mOsm (PorousMP). This yielded comparable MP size distributions and surface morphology for each polymer, as shown in Fig. S2. Porous MP cross sections were imaged to view the internal microstructure (Fig. 2C). The measurement of the inner occlusion diameter shows that PLGA–OH occlusion diameter is significantly increased as compared to PLGA–COOH MPs, while PLGA–NH_2_ inner occlusions are smaller (Fig. 2D). When accounting for the cross-sectional area, only PLGA–NH_2_ displays an increase in the density of inner occlusions (Fig. 2E).

### Degradation rates and pH evolution of various end-capped PLGA

**Fig. 3:**
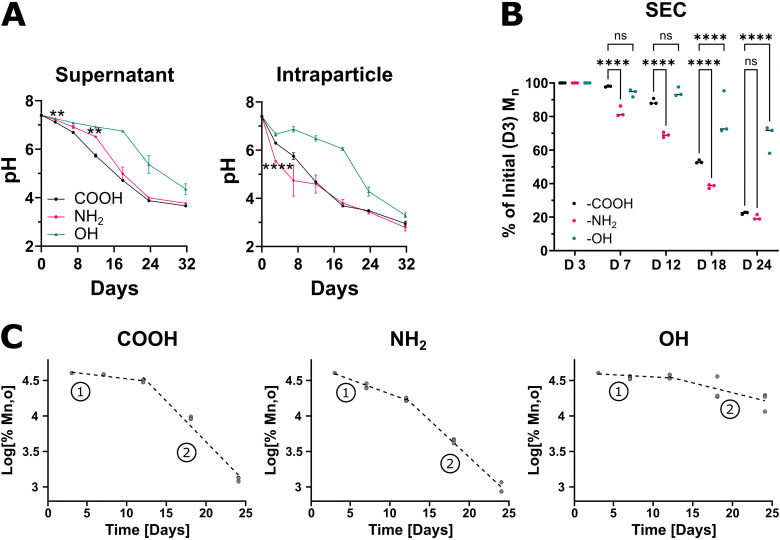
The rate of degradation and evolution of intraparticle pH is accelerated in amine functionalized PLGA. Porous microparticles formulated with end-capped PLGA were incubated in HEPES buffer to undergo degradation by hydrolysis. (A) Measurement of supernatant and intraparticle pH shows differential rates of acidification among the polymers. Notably, intraparticle pH rapidly reduces in PLGA–NH_2_, but the supernatant lags unlike the other end-capped PLGA. (B) Size exclusion chromatography data shows number averaged molecular weight reduces faster for PLGA–NH_2_. (C) log transformed SEC data was fitted to a linear degradation model (log_10_(%Deg.) = −k*t) for (1) non-catalytic erosion and (2) acid-catalyzed erosion phases of degradation. Degradation constants are in Table 1. Data represent mean ± sd for *N* = 3 samples. Statistical comparisons were made using two-way ANOVA followed by Tukey's multiple comparisons test. Comparisons shown in (A) are for PLGA–NH_2_ relative to PLGA–COOH. **p ≤ 0.01, ****p ≤ 0.0001.

We next investigated how various PLGA end-groups influenced polymer pH evolution and degradation kinetics. Intraparticle and supernatant pH evolution during porous MP degradation is quantified in Fig. 3A. The PLGA–OH demonstrated the slowest acidification (drop in pH) in both the supernatant and inside the MPs relative to the other formulations. Notably, for PLGA–NH_2_, the correlation between supernatant and intraparticle pH evolution deviates significantly from the other formulations. The intraparticle pH rapidly reduced but the supernatant pH took longer to decrease. In all formulations, the intraparticle pH and supernatant pH became indistinguishable from PLGA–COOH as the incubation time progressed (∼16 days for PLGA–NH_2_ and ∼32 days for PLGA–OH). Fig. 3B shows the degradation rate of the polymers as measured by % *M*_n_ relative to day 3 of degradation. Polymer molecular weight distributions of the degrading microparticles are shown in Fig. S3. To ensure full wetting of the polymer matrix, the measured *M*_n_ values normalized to the day 3 time point. Examining the molecular weights of the polymers revealed PLGA–OH produced the slowest degrading MPs while PLGA–NH_2_ MP showed a rapid reduction in *M*_n_ (Fig. 3B). A linear degradation model [log_10_(%Deg.) = −k*t] was used to quantify the rates for (1) initial non-catalytic erosion and (2) later acid-catalyzed erosion phases of degradation (Fig. 3C), as previously reported.^33^ The calculated degradation constants in Table 1 illustrate that *k*_1_ and *k*_2_ are depressed for PLGA–OH, while PLGA–NH_2_ has increased *k*_1_ relative to PLGA–COOH.

**Table 1:** End-capped PLGA degradation constants

Polymer	*k*_1_ (Days^−1^)	*k*_2_ (Days^−1^)
PLGA–COOH	0.013	0.115
PLGA–NH_2_	0.041	0.104
PLGA–OH	0.006	0.028

### Polymer end-cap differentially activates macrophages

**Fig. 4:**
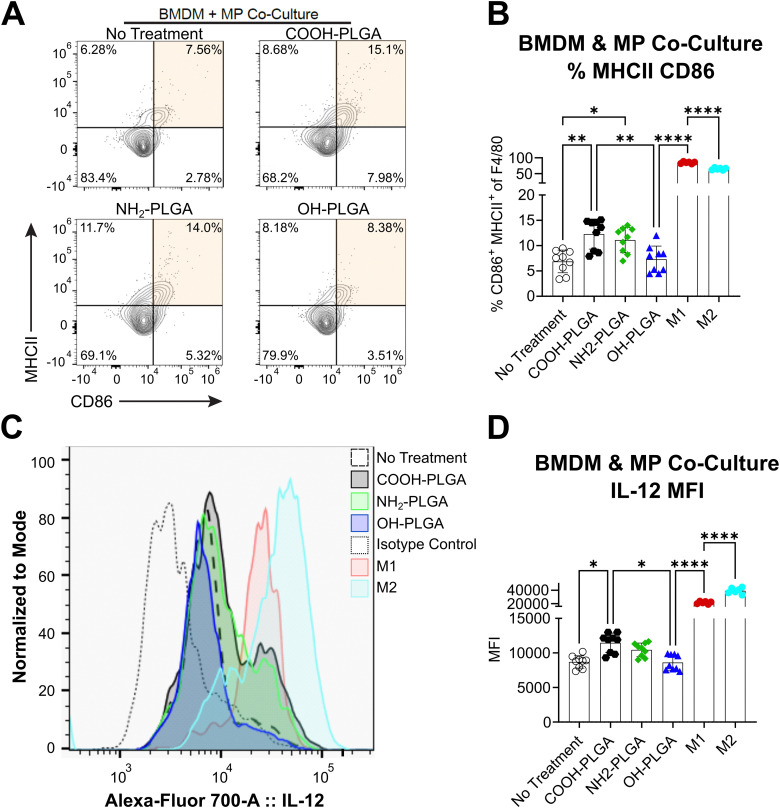
End-cap PLGA groups augment inflammatory macrophage differentiation and hydroxyl terminal groups are less immunogenic. Murine BMDMΦs and PLGA MPs were co-cultured for 3 days and analyzed by flow cytometry. Size distributions of MPs used for these assays are reported in Fig. S2B. (A) Flow plots of live CD11b^+^ F4/80^+^ BMDMΦs expressing MHCII and CD86. (B) Graph depicts frequency of double positive populations. (C) Histograms of IL-12 fluorescence and (D) calculated median fluorescence intensity (MFI). Data represents mean ± sd for *N* = 9 samples (*n* = 3 wells per mouse). Statistical comparisons were made using one-way ANOVA followed by Tukey's multiple comparisons test. *p ≤ 0.05, **p ≤ 0.01, ***p ≤ 0.001, ****p ≤ 0.0001.

Co-cultures with bone marrow-derived macrophages (BMDMΦs) and end-capped PLGA MP with diameter >15 μm (see Fig. S2B for particle size distribution) were conducted to determine if the observed changes to material properties affected cellular interactions. Large, phagocytosis resistant MP were selected for this experiment to simulate interactions that would occur for a formulation intended for long-term tissue residence. The gating threshold to separate microparticles and BMDMΦs is shown in Fig. S4. Markers for pro-inflammatory M1-like BMDMΦs were analyzed and it was observed that PLGA–COOH and PLGA–NH_2_ groups drove the differentiation of the BMDMΦs towards an activated, CD86^+^MHCII^+^ subset (Fig. 4A and B). Interestingly, BMDMΦs exposed to PLGA–OH MPs were phenotypically similar to the untreated, M0 control. IL-12 expression was elevated in BMDMΦs co-cultured with PLGA–COOH and PLGA–NH_2_ MP, while levels of IL-12 in BMDMΦs exposed to PLGA–OH were similar to the untreated controls (Fig. 4C and D). We compared the activation states induced by MPs to canonical lipopolysaccharide (LPS) & IFN-γ stimulated M1 controls, as well as canonical IL-4 stimulated M2 controls. Both controls exhibit an order of magnitude greater IL-12 expression and increased CD86^+^MCHII^+^ population frequency than all MP treated groups. Phenotypic marker expression for M1 (iNOS) and M2 (CD301b, CD206) are shown for all groups in Fig. S5.

### Polymer end-cap influences microparticle uptake and macrophage polarization

**Fig. 5:**
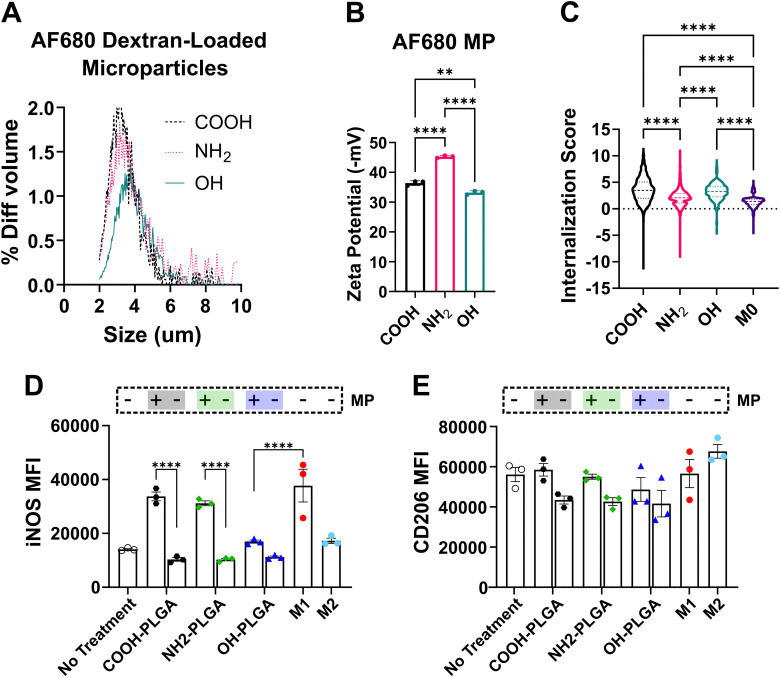
PLGA–NH_2_ microparticles exhibit reduced internalization by macrophages. Murine BMDMΦs and PLGA MPs were co-cultured for 24 hours and analyzed by ImageStream or spectral cytometry. (A) Microparticle size distribution and (B) zeta potential for microparticles formulated to contain Alexa fluor 680 labeled dextran. (C) ImageStream calculated internalization score, which is based on a ratio of the AF680 probe (MP) to cellular stain (CD11b) intensity. Flow cytometric quantification of (D) M1 phenotypic marker iNOS and (E) M2 phenotypic marker CD206 for BMDMΦs with internalized microparticles (+) as compared to BMDMΦs without internalized microparticles (−). Data in (A) represents the size distribution of 10 000 events for each formulation. Data in (B–E) represents the mean ± SD for *N* = 3 individual experiments. Data in (C) represent pooled data from *N* = 3 individual experiments. Statistical comparisons were made using one-way ANOVA followed by Tukey's multiple comparisons test. **p ≤ 0.01, ****p ≤ 0.0001.

To assess how different microparticle end-cap group chemistries may affect microparticle internalization, we performed an additional co-culture experiment with fluorescently labeled MPs with mean diameters of 3.23 ± 0.72 μm, 3.40 ± 0.90 μm, and 3.77 ± 0.85 μm for PLGA–COOH, PLGA–NH_2_, and PLGA–OH, respectively. The microparticle size distribution and zeta potential are reported in Fig. 5A and B, respectively. ImageStream analysis, which combines flow cytometry and imaging techniques, shows MPs made from PLGA–NH_2_ exhibited markedly reduced uptake by BMDMΦs as compared to PLGA–COOH and PLGA–OH MP (Fig. 5C). Representative images of internalized, labeled MPs can be found in Fig. S6. Flow cytometric quantification was performed to determine how MP internalization may affect BMDMΦ phenotypes. The fluorescent dextran encapsulated in the microparticles was used to identify populations of BMDMΦs that have internalized MP (AF680^+^) and those that have not internalized MP (AF680^−^). It was found that the AF680^+^ population of BMDMΦs had significantly elevated expression of the M1 marker, iNOS (Fig. 5D), and slightly elevated expression of the M2 marker, CD206 (Fig. 5E), relative to the AF680^−^ population for PLGA–COOH and PLGA–NH_2_. Notably, PLGA–OH did not induce significant changes of either marker.

### Variations in PLGA terminal group chemistry influence release kinetics

**Fig. 6:**
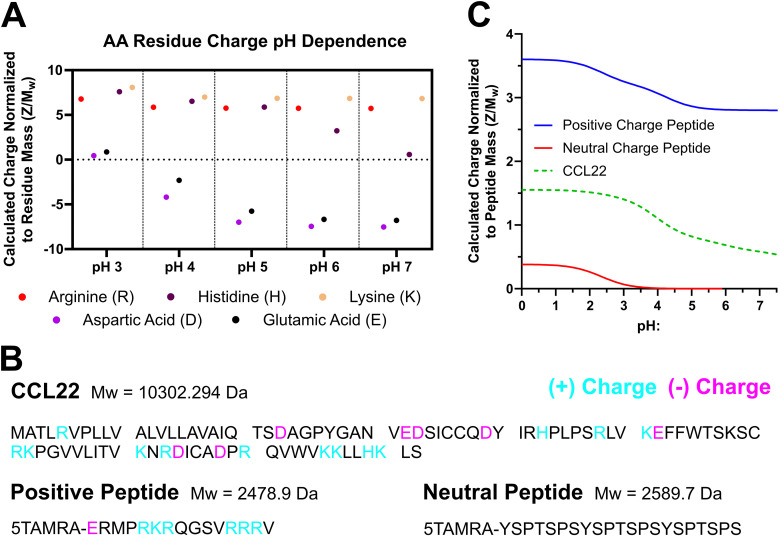
Five key amino acids are responsible for biomolecule net charge. Biomolecules encapsulated in PLGA MPs have potential to exhibit charge interactions with the polymer terminal groups. (A) Calculations of amino acid (AA) residue charge across typical supernatant and intraparticle pH ranges show negatively charged residues become uncharged in acidic conditions. Three biomolecules were selected for encapsulation: 2 peptides with positive and neutral charge and *M*_w_ ∼ 2.5 kDa and a more complex protein, CCL22, *M*_w_ ∼ 10 kDa. The sequences for these biomolecules are detailed in (B), and residues with potential for positive (blue) or negative (purple) charge are highlighted for each. (C) Net charge calculations were performed for these biomolecules from pH 0 to 7.

Biomolecules of various charge and size were encapsulated in PLGA microparticles to determine how the release rate may be affected by PLGA end-group chemistry. Fig. 6A details the molecular weight, lists the amino acid residues, and indicates residue charge at different pH. Table S1 details the calculated charge for all amino acids at pH 3–7. Three different biomolecules were encapsulated in end-capped PLGA microparticles: PKCε peptide substrate (∼2.5 kDa, positively charged peptide), CDK7tide (∼2.5 kDa, neutral peptide), and CCL22 (10 kDa, protein with net positive charge). Fig. 6B shows the amino acid sequences for each of these biomolecules and delineates the presence of charged residues. The calculated net charge of these biomolecules from physiologic to acidic pH is detailed in Fig. 6C. There is no overlap in biomolecule charge at any pH, and each shows a charge dependency on pH with full charge reached at pH < 2. Notably, the neutral peptide remains uncharged until pH 3, at which point it becomes weakly positive.

**Fig. 7:**
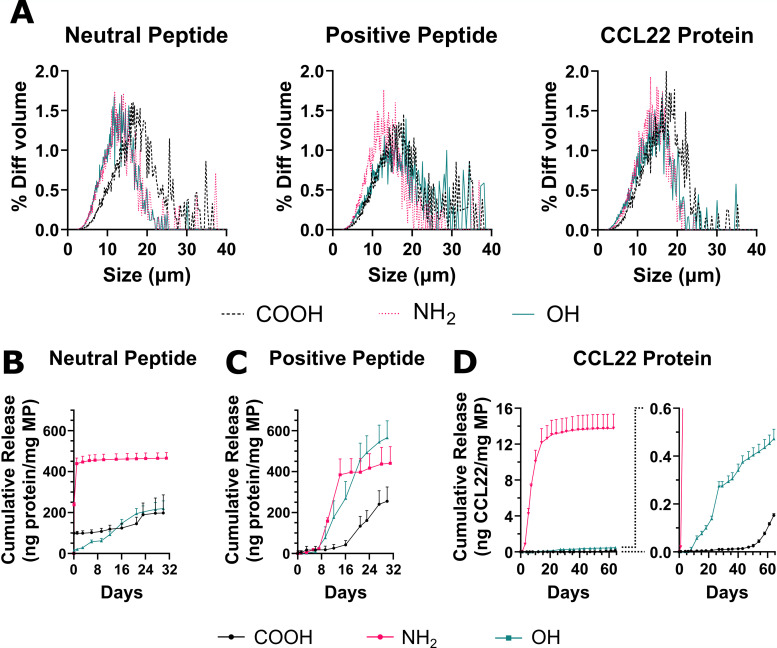
Biomolecule release & encapsulation with end-cap PLGA shows charge-dependent behavior. Cumulative release of positively and neutrally charged peptide from porous MPs with altered PLGA end caps was quantified. (A) Size distributions of microparticles loaded with the neutral peptide, positive peptide, and CCL22 protein. Table S2 details the mean and standard deviation of the size distribution for each formulation. (B) Neutral peptide release kinetics demonstrate biomolecule release in the absence of charge interactions. (C) Positively charged peptide release is also influenced by peptide–polymer electrostatic interactions. (D) For the larger and more complex protein, CCL22, the order of release is consistent with that for the positive peptide, but release from PLGA–OH and PLGA–COOH MPs is significantly depressed compared to PLGA–NH_2_ MPs. Data in A represent volume impedance measurement of particle size for *N* = 10 000 events. Data in (B)–(D) represent mean + sd for *N* = 3 independent release experiments.

Cumulative release profiles for these peptides from porous MP with different PLGA end caps reveals polymer-specific effects on release (Fig. 7). The size distributions for each MP formulation are shown in Fig. 7A, and the associated means and standard deviations are reported in Table S2. The release of the neutral peptide (Fig. 7B) from PLGA–NH_2_ MPs shows a monophasic burst release and release is concluded within 1 week. PLGA–OH MPs exhibit sustained release without a lag phase, and PLGA–COOH MPs have a triphasic (burst-lag-burst) release curve for the neutral peptide. For release of the positively charged peptide (Fig. 7C), all polymer formulations display a triphasic release profile (burst-lag-burst), though the duration of the lag phase differs considerably, lasting 7, 9, and 16 days respectively for PLGA–NH_2_, PLGA–OH, and PLGA–COOH MPs. Notably, PLGA–OH and PLGA–NH_2_ MPs release faster during the secondary burst phase than PLGA–COOH MPs. To determine if these charge effects apply to a larger, more complex biomolecule with both positively charged and negatively charged residues, release profiles were determined for the CCL22 protein (Fig. 7D). Similar to the positively charged peptide, release of CCL22 begins early from PLGA–NH_2_ MPs (1 day), followed by PLGA–OH MPs (7 days), and later from PLGA–COOH MPs (50 days). CCL22 release kinetics from PLGA–OH MPs resemble a linear profile, similar to the positively charged peptide, but the magnitude of release is depressed by 2 orders of magnitude relative to PLGA–NH_2_ MPs. This differs considerably from the trends observed in the positively charged peptide. Interestingly, end-capped PLGA MPs encapsulated with CCL22 have greater surface porosity for COOH and OH MPs but reduced pores for NH_2_ MP, as compared to the peptide formulations (Fig. S7).

## Discussion

The present study examines how changing the PLGA terminal group affects polymer physical properties and release kinetics for encapsulated peptides or protein. While PLGA with carboxylic acid (COOH), ester (COOCH3), and hydroxyl (OH) groups have been previously explored,^4,6,16,19,22,34–37^ this is the first report, to our knowledge, of material properties and release kinetics for PLGA terminated with an amine (NH_2_) group compared to other end-group chemistries. Because PLGA is extensively used to develop long-acting injectable formulations,^4,8,38,39^ we endeavored to conduct rigorous testing of polymer physical properties, degradation, phagocytic uptake of formed microparticles, and biomolecule release. We demonstrate that PLGA polymer terminated with an amine group (PLGA–NH_2_) reduces the resultant polymer hydrophobicity compared to PLGA terminated with carboxylic acid (PLGA–COOH) or hydroxyl (PLGA–OH) groups. We also observed double emulsion microparticles (MP) manufactured from PLGA–NH_2_ exhibit a reduced inner occlusion diameter and increased occlusion density, which is consistent with a more stable inner emulsion. Furthermore, the degradation rate of PLGA with NH_2_ end-capping was significantly accelerated, which may be related to the polymer material properties (*i.e.* hydrophilicity). Likewise, the fast degradation leads to more rapid evolution of acidic pH inside the MP. These findings that terminal group can dictate polymer physical properties are consistent with prior work characterizing differences between COOCH3, COOH, and OH terminated PLGA.^34–37^ We also note that PLGA–NH_2_ has reduced phagocytic uptake and that PLGA–OH has reduced potential to be immunogenic. Additionally, PLGA terminal groups trigger different release kinetics of both small peptide (*M*_w_ = 2.5 kDa) and larger protein (*M*_w_ = 10 kDa) biomolecules. Prior studies have investigated how variations in PLGA terminal groups alters the release rates of small molecule drugs.^35–37^ We also previously evaluated how acid and ester terminated PLGA of variable molecular weights modulate the release rates of small, charged peptides.^19^ Interestingly, our data suggest biomolecules encapsulated from PLGA–NH_2_ have accelerated release rates. Further, we observed that the rate of release is dependent on the biomolecule charge and is not purely a consequence of more rapid polymer matrix erosion or swelling, as is the case for small molecule release.

Prior studies have investigated how the inclusion of functional groups or modification of terminal groups can affect the crystal structure of a polymer. For instance, backbone modification directly affects the crystallinity of a polymer^40^ and has the potential to produce highly ordered crystal structures that are conductive.^41^ Prior work has also shown the size of a terminal group can reduce the rate of crystallization for polyethylene oxide^42^ and polycaprolactone^43^ polymers, in which tested terminal groups included a 60-carbon chain and addition of aromatic rings (phenyl, naphthyl, anthracenyl groups), respectively. Of note, polycaprolactone terminated with an anthracenyl group produced the most pronounced effect, while the phenyl and naphthyl groups were less modulatory suggesting a critical size limit.^43^ Our DSC analysis of PLGA–COOH, PLGA–OH, and PLGA–NH_2_ polymers revealed PLGA–NH_2_ has reduced *T*_g_ compared to PLGA–OH and PLGA–COOH, suggesting the crystal structure may be altered. However, because there are no backbone modifications in these polymers and the size of the terminal groups are small, alternative explanations should be considered. Prior reports indicate that water absorbed into the polymer matrix acts as a plasticizer, which can result in decreased *T*_g_.^34^ Further, we found that PLGA–NH_2_ exhibits greater hydrophilicity, as measured by reduced water droplet surface tension, relative to PLGA–COOH and PLGA–OH. Thus, it is plausible that the measured *T*_g_ of PLGA–NH_2_ is reduced due to PLGA–NH_2_ being more hygroscopic as opposed to altered crystallinity.

To understand the effects PLGA–NH_2_ has on MP formation and surface morphology, we initially formulated double emulsion MPs with ultra-pure de-ionized water for the inner aqueous phase, which we expected to yield spherical particles without surface porosity. Surprisingly, we observed some surface pores on MP formulated from PLGA–COOH, and much more with PLGA–NH_2_. Notably, we observed an increase in osmolarity after incubating PLGA–NH_2_ in water, compared to PLGA–COOH or PLGA–OH. This suggests the water-soluble fraction of low *M*_w_ PLGA from the PLGA–NH_2_ polymer, in particular, may act as an osmolyte to facilitate pore formation,^26,44,45^ which could contribute to the increased porosity of the PLGA–NH_2_ MPs, even when salt is not added to the inner aqueous phase. In order to ensure consistent morphology with each polymer, we next formulated MPs to be pre-populated with surface pores (porous MP) for polymer degradation and biomolecule release experiments. This is accomplished by changing the osmolarity of the inner aqueous phase of the emulsion to generate an osmotic gradient. Pre-formation of pores also reduces the physical barriers to diffusion through the polymer matrix, which has been shown to accelerate release of proteins from polymeric MP.^27^ Porous MP had consistent surface morphology among the end-capped PLGA, but analysis of MP cross-sections revealed MP formulated with PLGA–NH_2_ have smaller inner occlusion diameters and a greater density of occlusions. This result suggests the inner emulsion may be more stable during PLGA–NH_2_ MP formation. The terminal NH_2_ groups with a p*K*_a_ of 4–5 would exist in protonated and charged form (NH_3_^+^) during MP formation. In addition to contributing to the osmotic gradient and MP porosity, low molecular weight positively charged PLGA–NH_2_ dissolved in the inner aqueous phase may also act as an amphiphilic surfactant, which can reduce interfacial surface tension^46^ to yield a more stable inner emulsion.

Porous MP were selected for all subsequent experiments due to prior work documenting their ability to accelerate biomolecule release.^26^ Our experiments to investigate MP degradation showed differential rates of degradation among the different end-capped PLGA. PLGA–NH_2_ had greater rate of reduction in *M*_n_, while PLGA–OH *M*_n_ decreased slower, compared to PLGA–COOH. In all polymer MPs, we observed a two-phase degradation process, which is consistent with prior work characterizing the degradation of other PLGA.^33,47^ Initial uncatalyzed hydrolysis of the polymer matrix (phase 1) is followed by more rapid, acid-catalyzed hydrolysis of the polymer's ester linkages (phase 2). The phase 1 degradation constant associated with non-catalytic hydrolysis for PLGA–NH_2_ is greater than those for PLGA–COOH and PLGA–OH. Many factors can influence degradation rate including increased polymer solubility, absorption of water molecules into the polymer matrix that break the ester bonds, and local pH.^15,17,33,48^ Indeed, our data show PLGA–NH_2_ is more hydrophilic and that it is possible that low molecular weight oligomers readily solvate into the supernatant than for PLGA–COOH or PLGA–OH. Further, PLGA–NH_2_ demonstrated the most rapid reduction in intraparticle pH during the first 12 days of degradation, consistent with the observed faster polymer degradation rate, and therefore, faster accumulation of acidic (COOH) end groups. After that point, intraparticle pH for PLGA–NH_2_ and PLGA–COOH are similar, as the evolving COOH end groups become dominant due to polymer hydrolysis. Interestingly, PLGA–NH_2_ supernatant pH did not show a proportional decrease to intraparticle pH changes occurring in the early timescale. Our data suggest that the initial PLGA–NH_2_ polymer is more hydrophilic (lower contact angle) and more water soluble (greater osmolarity after incubation in water). Therefore, solubilized PLGA–NH_2_ segments present in the supernatant at early time points may act as H^+^ scavengers *via* the NH_2_ terminal group, thereby buffering and minimizing the initial drop in supernatant pH. Unlike PLGA–NH_2_, PLGA–OH shows significantly reduced degradation rates and pH evolution relative to PLGA–COOH despite comparable material properties (*i.e.*, WCA and *T*_g_).

Variations in polymer physical properties and microparticle degradation may potentially alter how phagocytes interact with and internalize MPs. In our first experiment, we demonstrated that large (>15 μm), phagocytic resistant particles increase BMDMΦ expression of the M1 marker iNOS, albeit to a much lower level compared to canonical stimuli, and does not increase expression of M2 phenotypic markers. Our data shows differential expression of macrophage markers of activation including CD86, MHCII, and IL-12 among the end-capped PLGA. Specifically, PLGA–OH MPs did not lead to an increase in the activated, CD86^+^MHCII^+^ subset or elevated expression of IL-12, while PLGA–COOH and PLGA–NH_2_ produced comparable increases. This suggests that the differential levels of activation and IL-12 expression may be due to a state of frustrated phagocytosis, which has been reported to increase macrophage activation and cytokine production in studies investigating the uptake of different sized β-glucans.^49^ This finding prompted our subsequent, microparticle internalization study of small (<5 μm) particles.

Phagocytosis of small particles has been extensively studied^50,51^ and is highly dependent on particle physical properties including hydrophobicity, size, and surface chemistry, among others. For instance, poly(ethylene glycol) (PEG) terminated PLGA nanoparticles exhibit reduced phagocytosis due to the PEG forming a hydrated shell, resulting in extended circulation.^52^ In general, more hydrophobic particles are known to be more readily phagocytosed.^53^ Additionally, particle charge can affect phagocytosis.^51^ Our analysis of the microparticle zeta potential revealed all microparticles to be negatively charged (between −30 and −45 mV). The greater negative charge density on the PLGA–NH_2_ MP is likely a result of greater encapsulation of the negatively charged Alexafluor680 labeled dextran. In our internalization study, we observed reduced macrophage phagocytosis of PLGA–NH_2_ MP relative to the phagocytosis of PLGA–OH and PLGA–COOH MP. Our work characterizing the material properties of PLGA–NH_2_ indicates that it is more hydrophilic than the other two PLGA polymers, thus it should be less readily phagocytosed. Further, PLGA–NH_2_ MPs may form a hydration shell, similar to PEG terminated PLGA, resulting in the observed reduction to phagocytic clearance. Despite reduced internalization rates, we show that internalization of PLGA–NH_2_ MP causes macrophages to increase expression of the M1 associated marker, iNOS, and that this expression is comparable to the M1 control value. PLGA–COOH MP also causes increased iNOS expression following internalization, but PLGA–OH MP do not. This difference in activation state upon internalization may be a result of the reduced surface charge density of PLGA–OH MP as well as its greater hydrophobicity – both of which have been shown to modulate particle internalization.^54^

Together, these experiments suggest that MP terminal groups can influence the phagocytic capacity of macrophages and influence markers of macrophage activation. One implication of this finding is that it may be possible to utilize different end group chemistries on PLGA to amplify immune-targeting therapeutics. For instance, IL-12 is a potent cytokine that has been shown to be critical for effector T cell induction, which is required to reject tumors.^55,56^ Thus, PLGA–COOH MP, which exhibited the largest increase in IL-12 expression and the ability to skew naïve BMDMΦs towards an M1 activated state, may provide a synergistic effect in cancer treatments. Conversely, therapeutic modalities that intend to minimize the activation of macrophages may find additional benefit from formulations that use PLGA–OH. Further investigation will be needed to determine if terminal groups impact the phagocytosis and activation state of other immune cells such as neutrophils, dendritic cells and monocytes, and to determine if these findings have implications for *in vivo* applications.

One of the most common *in* vivo applications of polymeric microparticles is to enable sustained release of small molecules or biomolecules for several weeks to months.^39^ Our data shows MP formulations containing CDK7tide (neutral peptide), PKCε peptide substrate (positive peptide), or a larger 10 kDa therapeutic protein (CCL22) show significant differences in their release profiles. The neutral peptide release shows how a small peptide (*M*_w_ = 2.5 kDa) releases from different end-capped PLGA MP in absence of electrostatic interactions. Both PLGA–COOH and PLGA–NH_2_ exhibit a large, initial burst release upon hydration and swelling of the polymer matrix while PLGA–OH shows a more controlled, linear release profile. The slow degradation of PLGA–OH and its terminal group remaining uncharged, reducing its interaction with water molecules relative to PLGA–COOH and PLGA–NH_2_, likely temper the burst release. Release of the positively charged peptide, however, exhibits lag periods lasting 16, 6, and 7 days for PLGA–COOH, PLGA–NH_2_, and PLGA–OH, respectively. Since the neutral and positive peptide are the same size (*M*_w_ = 2.5 kDa), this delay in release of the positive peptide may be attributed to charge interactions between the peptide and polymer matrix. Because MP formulation occurs in the presence of water and hydrolysis may occur to some extent during this process, we normalized the degradation data to day 3 *M*_n_ values for comparisons due to observed deviations. However, this early degradation causes COOH terminal groups to form in both the slow degrading PLGA–OH and fast degrading PLGA–NH_2_. With some COOH terminal groups to bind to, the positively charged peptide release is slowed, though not to the same degree as PLGA–COOH resulting in the observed release profiles. This is consistent with our group's prior observation that the release of cationic peptides is dependent upon the frequency of charged terminal groups available to form electrostatic interactions.^19,22^ Additionally, PLGA–NH_2_ and PLGA–OH begin their secondary burst at comparable times suggesting similar levels of hydrolysis during MP formulation. Notably, however, release continues to day 32 for PLGA–OH but only to day 12 for PLGA–NH_2_. For PLGA–NH_2_, our data shows the intraparticle pH is ∼4.6 by day 12 (equal to PLGA–COOH) and coincides with polymer *M*_n_ degraded to 70% of day 3 values liberating significant COOH terminations. In addition, the positively charged peptide becomes even more positively charged below pH 5. Taken together, the halt in positively charged peptide release from PLGA–NH_2_ at day 12 is likely a result of greater electrostatic interaction strength and frequency within the polymer. Likewise, PLGA–OH does not reach comparable intraparticle pH and degradation until day 24, matching a slight reduction in the release rate at a similar time. While the polymer terminal group most significantly modulated the lag phase of release for the positively charged peptide, the shapes of the release curves were largely conserved between each end-cap group. CCL22 releasing MP formulations exhibit a similar trend in the lag phase duration as positively charged peptide releasing MPs. PLGA–NH_2_ MPs begin releasing first (day 1), followed by PLGA–OH MPs (day 8) and then PLGA–COOH MPs (day 48). Following the lag, CCL22 release kinetics from PLGA–COOH and PLGA–OH resemble positively charged peptide release kinetics, including a slowing of CCL22 release at day 24 from PLGA–OH MP. This would suggest that electrostatic interactions regulate the release of the positively charged peptide and CCL22 protein. However, compared to the peptide, CCL22 release from PLGA–NH_2_ MPs differs considerably, suggesting there may be additional differences in underlying electrostatic interactions between the polymer and biomolecules.

The present study provides insights into how PLGA terminal group chemistry can affect biomolecule release. One important limitation of this manuscript is that only three different end-capped PLGA polymers were explored. In the past two decades, the repertoire of commercially available PLGA has increased to now include NHS-ester, thiol, azide, maleimide, and poly(ethylene glycol), among others commonly used to functionalize PLGA nanoparticles,^57,58^ all of which possess different potential for biomolecule interaction. Further work examining the effects of terminal groups beyond our existing understanding of COOH, COOR, OH, and our new understanding of NH_2_ are merited, especially considering how drastically the physical properties changed for PLGA–NH_2_. Additionally, this manuscript focused on low molecular weight PLGA and did not consider higher molecular weight PLGA. As the molecular weight increases, the frequency of terminal groups will decrease while the polymer crystallinity increases, leading to greater hydrophobicity. This will likely mute the differences between end-capped PLGA. Thus, it is possible that there is a critical molecular weight required to achieve the observed changes in material properties in modulation of biomolecule release. Future work may describe and better understand this critical parameter. Furthermore, biomolecule release from PLGA–NH_2_ and PLGA–OH strongly suggests that electrostatic driven interactions between the polymer and biomolecular cargo have been altered. Because electrostatic interactions often precede acylation reactions at acidic pH within the degrading microparticles,^20–22^ PLGA–NH_2_ or PLGA–OH may also alter protein acylation. Thus, future work expanding this study to include an investigation of protein sorption and subsequent acylation on PLGA surfaces with different densities of NH_2_ groups would be instrumental in understanding the exact mechanism for the observed change to biomolecule release.

## Conclusions

Small alterations in PLGA chemical composition, specifically adjusting the terminal group chemistry, have the potential to cause significant changes to long-acting injectable formulations that deliver biomolecules. First, the terminal groups can alter polymer physical properties that control the formation and degradation of the polymer matrix. This work also shows that the PLGA terminal group chemistry affects phagocytic uptake of formed MPs and has the potential to alter macrophage activation state. Furthermore, altering the PLGA terminal group modulates the release of both charged and un-charged biomolecules. More specifically, our data suggests altering the PLGA terminal group modulates electrostatic interactions between the polymer matrix and positively charged biomolecules. Further studies are merited to better characterize these charge interactions throughout the degradation of the encapsulating polymer and how they may present with biomolecules of increasing complexity. Overall, the data generated in these studies provide insight into how changes in the PLGA terminal group chemistry could be leveraged as an additional design consideration for polymeric platforms delivering biomolecules.

## Author contributions

Matthew A. Borrelli – conceptualization; data curation; formal analysis, investigation, methodology, visualization, writing – original draft, writing – review & editing. Jordan J. P. Warunek – investigation, conceptualization, formal analysis, writing – review & editing. Tarini Ravikumar – investigation. Stephen C. Balmert – validation, writing – review & editing. Steven R. Little – funding acquisition, resources, supervision, writing – review & editing.

## Conflicts of interest

There are no conflicts to declare.

## Supplementary Material

TB-013-D5TB00816F-s001

## Data Availability

The matlab code associated with the peptide charge calculations presented in Fig. 6 has been added to this manuscript as a SI. All other data associated with this manuscript is maintained on an internal data repository at the University of Pittsburgh. Data will be made available upon reasonable request to the corresponding author. Expanded characterization of the end-capped PLGA polymers and the formed microparticles, additional flow cytometric characterization of co-cultured macrophages, and representative images of particle morphology and internalization by macrophages, among other information. See DOI: https://doi.org/10.1039/d5tb00816f
