# Supplementary material for: End group chemistry modulates physical properties and biomolecule release from biodegradable polyesters
Source: J Mater Chem B. 2025 Aug 6;13(34):10621–34. doi: 10.1039/d5tb00816f (PMC12338031; doi:10.1039/d5tb00816f)
Supplement: TB-013-D5TB00816F-s001 [file TB-013-D5TB00816F-s001.pdf]

Supplementary Information (SI) for Journal of Materials Chemistry B.
This journal is © The Royal Society of Chemistry 2025

Supplementary Table 1 – Calculated Charge for Amino Acid Residues

	pH 3	pH 4	pH 5	pH 6	pH 7
**Alanine (A)**	2.01480	0.24028	0.02427	0.00016	-0.02262
**Arginine (R)**	6.77090	5.86330	5.75280	5.74050	5.72880
**Asparagine (N)**	1.35870	0.16203	0.01637	0.00011	-0.01526
**Aspartic Acid (D)**	0.43732	-4.19590	-6.98920	-7.45880	-7.52260
**Cysteine (C)**	1.48150	0.17630	0.01399	-0.03831	-0.38543
**Glutamic Acid (E)**	0.85818	-2.30090	-5.75580	-6.67780	-6.79830
**Glutamine (Q)**	1.22830	0.14648	0.01480	0.00010	-0.01379
**Glycine (G)**	2.39120	0.28517	0.02881	0.00019	-0.26852
**Histidine (H)**	7.59560	6.51930	5.87310	3.22260	0.57293
**Isoleucine (I)**	1.36840	0.16320	0.01649	0.00011	-0.01537
**Leucine (L)**	1.36840	0.16320	0.01649	0.00011	-0.01537
**Lysine (K)**	8.06830	6.98690	6.85520	6.84030	6.82460
**Methionine (M)**	1.20300	0.14347	0.01449	0.00010	-0.01351
**Phenylalanine (F)**	1.08660	0.12959	0.01309	0.00009	-0.01220
**Proline (P)**	1.55910	0.18594	0.01878	0.00013	-0.01751
**Serine (S)**	1.70800	0.23070	0.02058	0.00014	-0.01918
**Threonine (T)**	1.50690	0.17971	0.01815	0.00012	-0.01692
**Tryptophan (W)**	0.87894	0.10482	0.01059	0.00007	-0.00987
**Tyrosine (Y)**	0.99069	0.11814	0.01189	-0.00039	-0.01582
**Valine (V)**	1.53230	0.18274	0.01846	0.00013	-0.01721

Supplementary Table 2 – Size distribution mean and SD of Biomolecule End-Cap PLGA Formulations

	COOH	NH_2	OH
**Prok-051**	μ = 16.32 μm<br>SD = 5.041 μm	μ = 13.08 μm<br>SD = 4.001 μm	μ = 16.28 μm<br>SD = 5.887 μm
**Prok-056**	μ = 17.17 μm<br>SD = 5.146 μm	μ = 12.96 μm<br>SD = 3.797 μm	μ = 12.98 μm<br>SD = 3.918 μm
**CCL22**	μ = 16.82 μm<br>SD = 4.827 μm	μ = 13.96 μm<br>SD = 3.911 μm	μ = 14.5 μm<br>SD = 4.495 μm

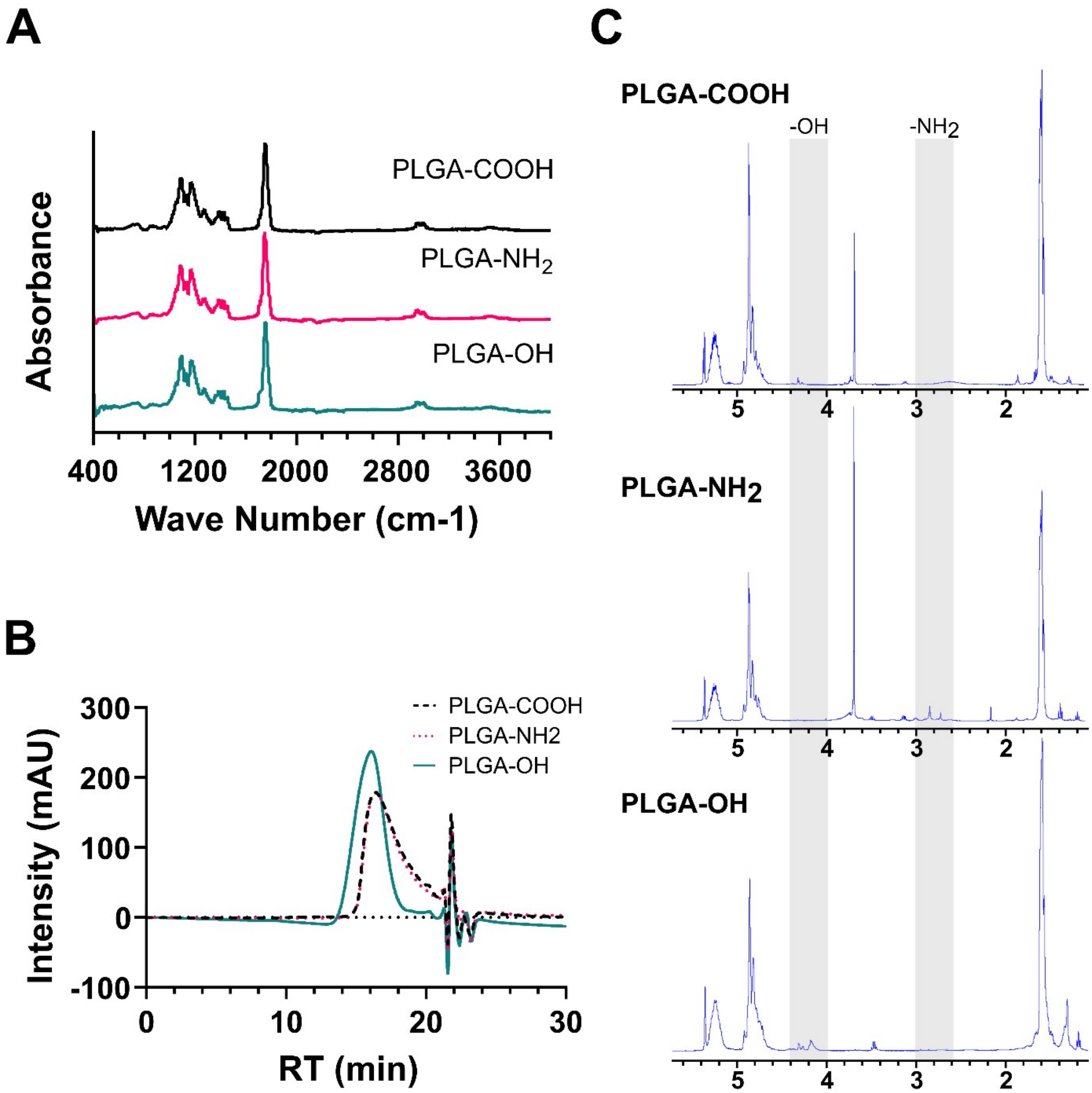

Supplementary Figure 1. Additional Material Characterization of End-Capped PLGA. (**A**) ATR-FTIR emission spectra, (**B**) molecular weight distribution, and (**C**) H-NMR emission spectra for end-capped PLGA polymers.

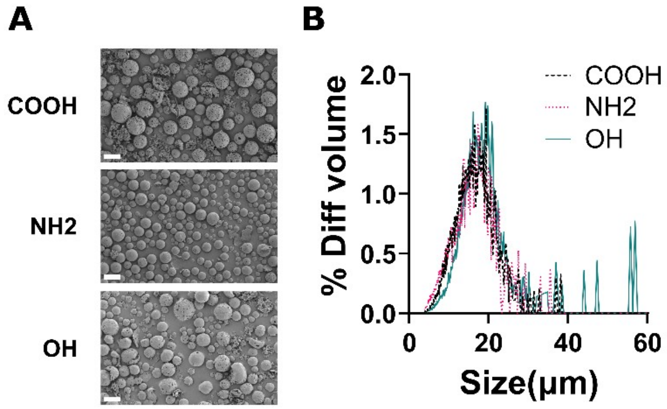

Supplementary Figure 2 – End-Cap PLGA Microparticle Morphology and Size

(**A**) Scanning electron microscopy images of porous, end-cap PLGA MP reveal a polydisperse size and the formation of surface pores on all MP formulations. Scale bar = 20 μm, 500x magnification. (**B**) Sizing data shows microparticles have consistent size distributions with mean diameters of 16.56 ± 4.86 μm, 16.50 ± 5.15 μm, and 17.86 ± 4.27 μm for PLGA-COOH, PLGA-NH_2, and PLGA-OH, respectively. (**C**) Incubation of PLGA-NH_2 MP with fluorescamine shows NH_2 terminal groups are present after MP fabrication. (**D**) All formulations of formed microparticles possess negatively charged zeta potential, in which PLGA-OH MP possess the least charge density. Statistical comparisons were made using one-way ANOVA followed by Šídák's multiple comparisons test * $p \leq 0.05$, **** $p \leq 0.0001$.

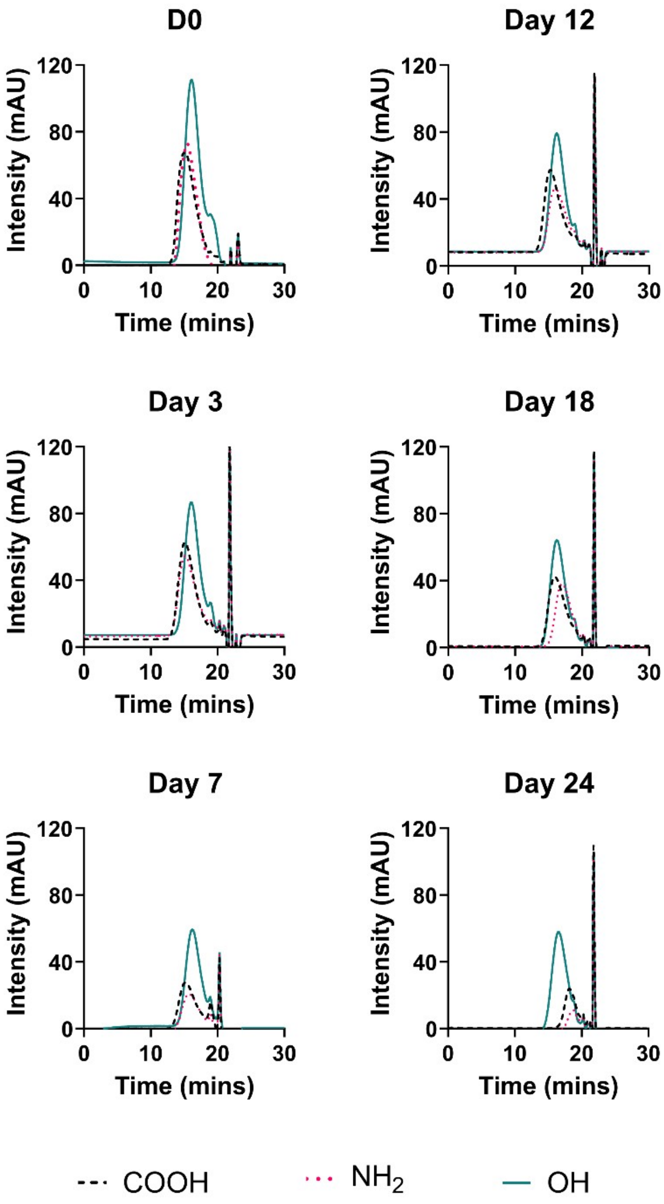

Supplementary Figure 3 – Molecular Weight Distributions of End-Capped PLGA During Degradation

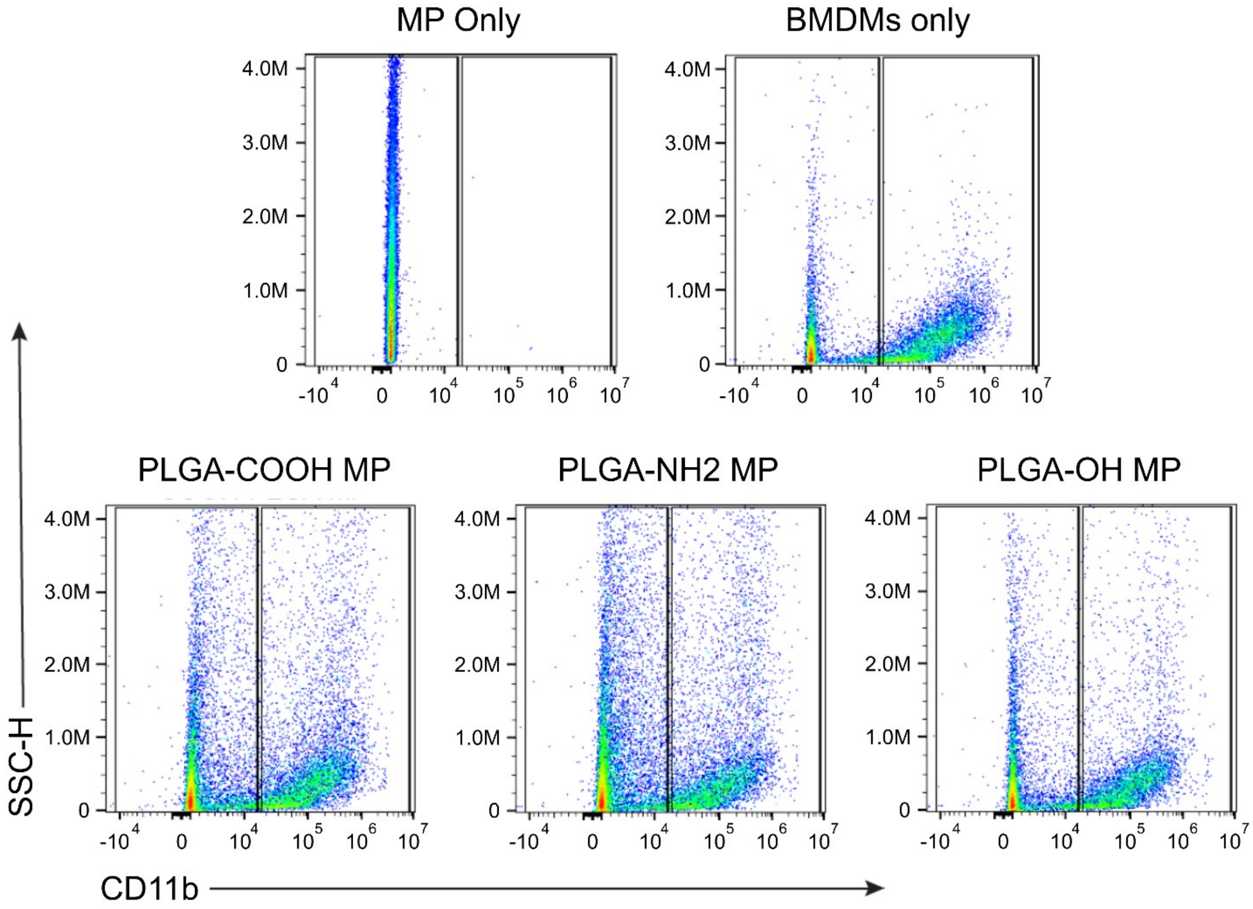

Supplementary Figure 4 – Gating strategy to quantify microparticle uptake by macrophages

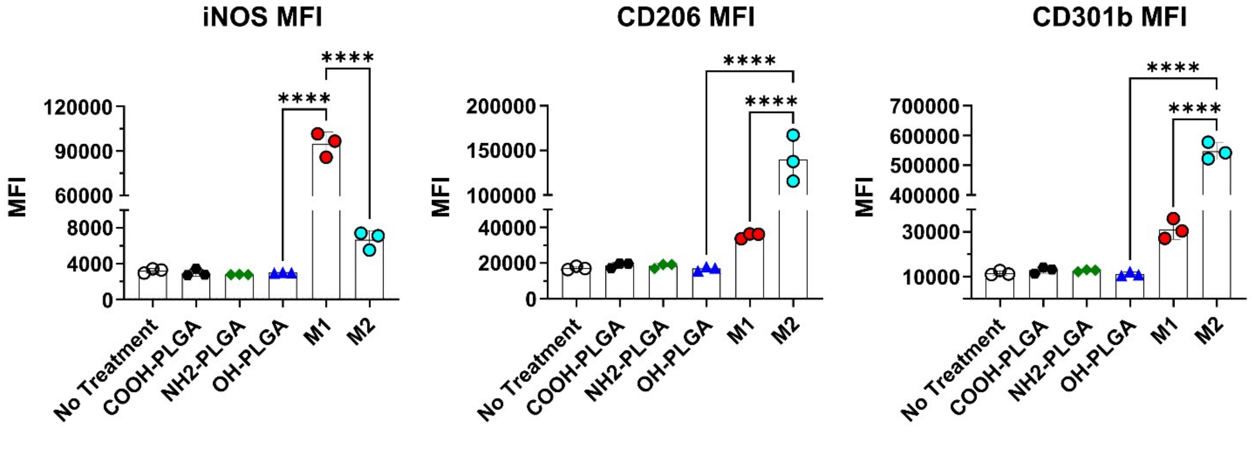

Supplementary Figure 5 – Mean fluorescent intensity of M1 and M2 phenotypic markers following incubation with large, phagocytosis resistant MP

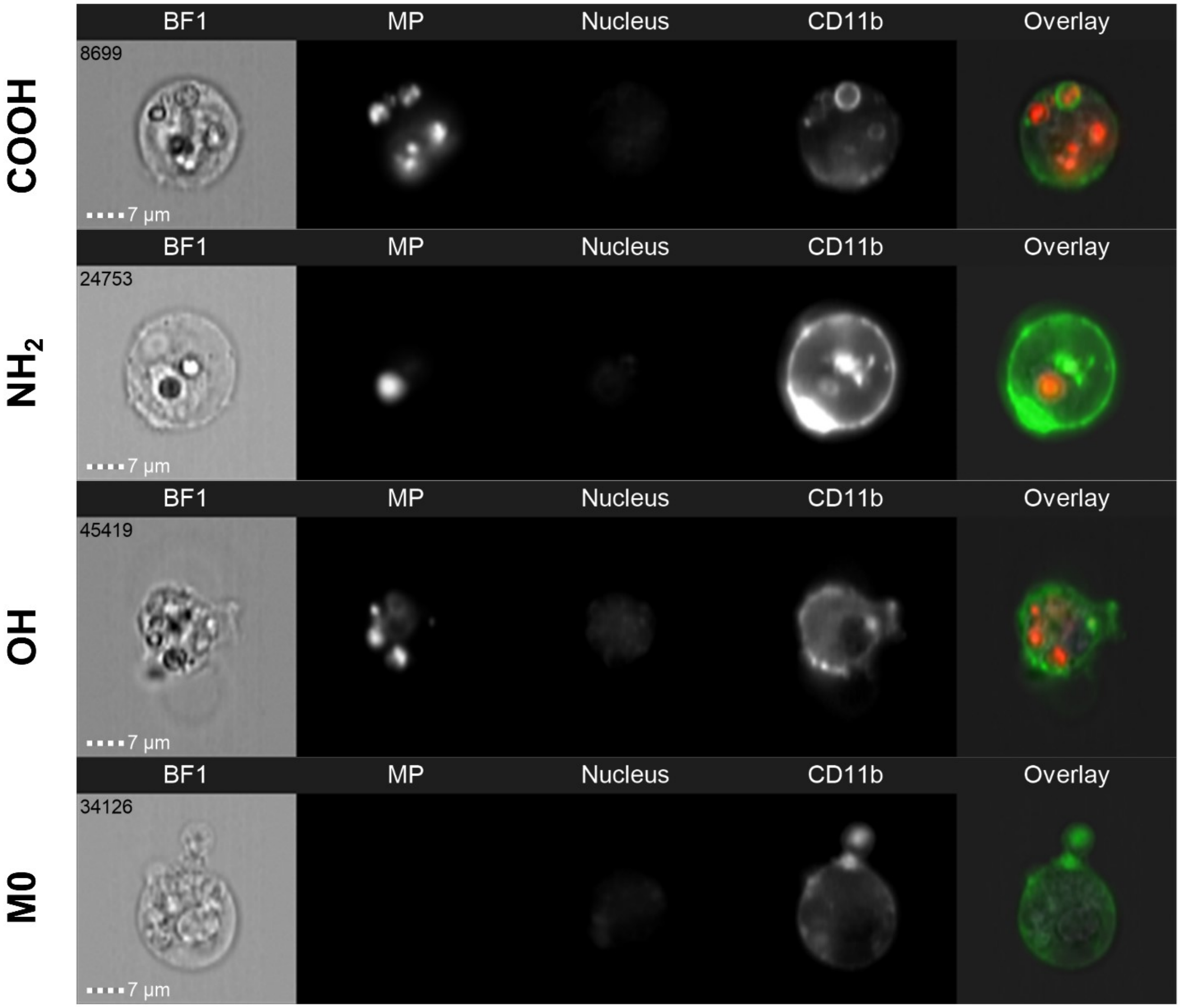

Supplementary Figure 6 – Representative images of microparticles internalized by BMDMΦs

Bright field microscopy 1 (BF1) shows cell morphology and internalized microparticles. Fluorescent intensity is shown for internalized microparticles (MP) loaded with Alexa Fluor 680 labeled dextran (681/704; ex/em), the nucleus (DAPI), and the cell membrane via CD11b staining (FITC). M0 BMDMΦs were cultured in the absence of MP, serving as a negative control. A colored overlay image is shown with the following weight to each image: BF1 – 20%, Nucleus – 80%, CD11b – 100%, MP – 100%.

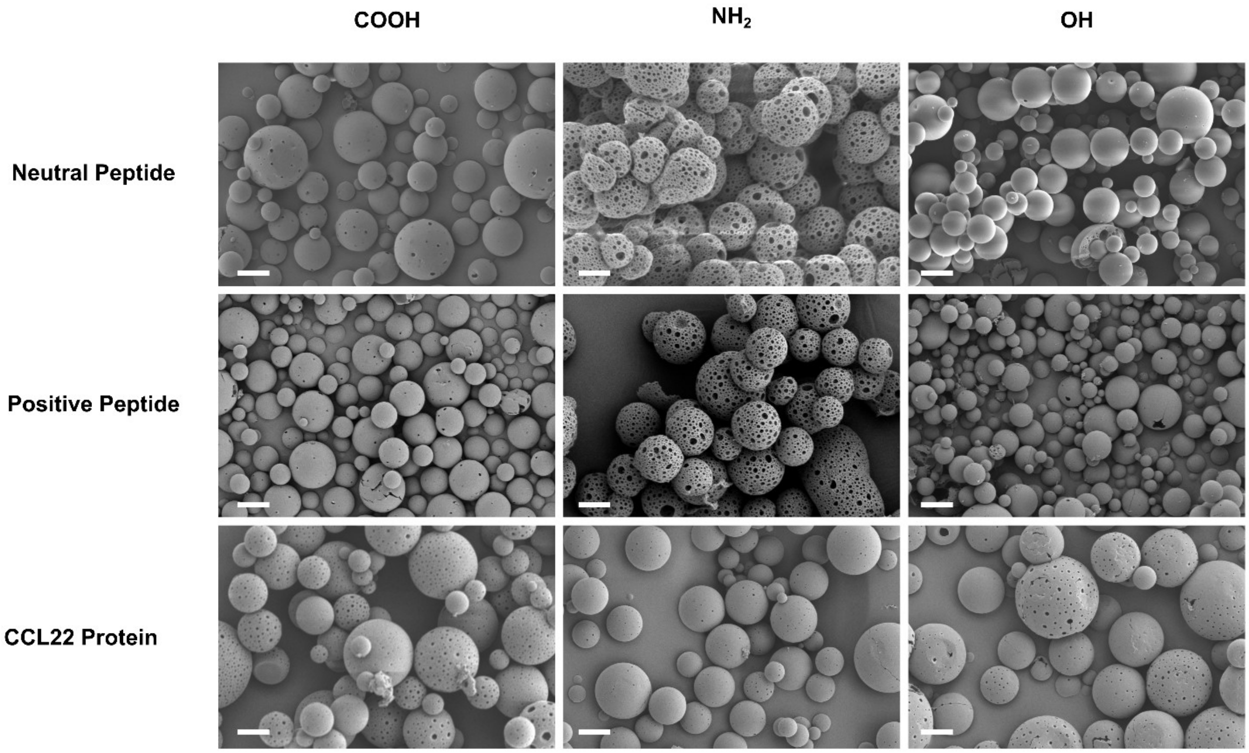

Supplementary Figure 7 – Scanning Electron Micrographs of End-Capped PLGA Loaded with Biomolecules

All images were captured at 1000x magnification, scale bar = 10 μm. All microparticles show porous surface morphology but variable frequency and size of surface pores. For the positive peptide and neutral peptide, NH_2 terminated PLGA produces highly porous microparticles while OH terminated PLGA produces less surface pores. An inverse effect to the surface pores frequency is observed for CCL22 protein loaded microparticles, in which PLGA-NH_2 MP exhibit less surface porosity.

Matlab Code for Peptide Charge Calculation

```
%Peptide_Charge_Calculator

clear; clc;

%AA Properties listed for AA's [A B C...]
AA_MW = [71.0788 0 103.1448 115.0886 129.1155 147.1766 57.052 137.1412 113.1595 0 128.1742 113.1595 131.1986...
    114.1039 0 97.1167 128.1308 156.1876 87.0782 101.1051 0 99.1326 186.2133 0 163.1760 0];

%AA ID = [A - C    D    E    F G H    I - K     L M N - P Q R     S T - V W - Y     -];
AA_pKa = [0 0 8.33 3.86 4.25 0 0 6.00 0 0 10.53 0 0 0 0 0 0 12.48 0 0 0 0 0 0 10.07 0];

AA_hydrophilicity = [-0.5 0 -1.0 3.0 3.0 -2.5 0 -0.5 -1.8 0 3.0 -1.8 -1.3 0.2 0 0 0.2 3.0 0.3 -0.4 0 -1.5 -3.4 0 -2.3 0];
%NEGATIVE = HYDROPHOBIC, POSITIVE = HYDROPHILIC


name = input('Enter Peptide Name: ','s');
sequence = input('Enter AA Sequence (rgd or RGD): ','s');
Labeled = input('Is the peptide labeled? (y/n) ','s');

%convert sequence to string of numbers -> A=65, B=66, ... Z=90
sequence = sequence(~isspace(sequence));    %removes any spaces
seq=single(upper(sequence));

if Labeled == 'y'
    Peptide_MW = input('Enter total MW (peptide+label; Da): ');
elseif Labeled == 'n'
    Peptide_MW=[];
    for i=1:length(seq)
        Peptide_MW = [Peptide_MW,AA_MW(seq(i)-64)]; %#ok<AGROW>
    end
end
```

```
Total_MW = sum(Peptide_MW)+18.0153; %adds MW of H2O from N & C terminal ends
AA_Count = length(seq);

Peptide_pKa=[];
for i=1:length(seq)
   Peptide_pKa = [Peptide_pKa,AA_pKa(seq(i)-64)]; %#ok<AGROW>
end

Peptide_hydrophilicity=[];
for i=1:length(seq)
   Peptide_hydrophilicity = [Peptide_hydrophilicity,AA_hydrophilicity(seq(i)-64)];
%#ok<AGROW>
end

%Net charge calculation for each pH
charge=[];
Z=[];
j=1;
for p=0:0.1:14 %pH values
   for i=1:length(seq)
      if Peptide_pKa(i)==12.48||Peptide_pKa(i)==10.53||Peptide_pKa(i)==6.00
         charge(i)=10^Peptide_pKa(i)/(10^p+10^Peptide_pKa(i)); %#ok<*SAGROW>
      elseif
Peptide_pKa(i)==3.86||Peptide_pKa(i)==4.25||Peptide_pKa(i)==8.33||Peptide_pKa(i)==10.07
         charge(i)=-10^p/(10^p+10^Peptide_pKa(i));
      else
         charge(i)=0;
      end
   end
   Z(j)=sum(charge) + (10^9.69/(10^p+10^9.69)) - (10^p/(10^p+10^2.34));%2nd term for N-
terminus, 3rd term for C-terminus
   j=j+1;
end
```

```
p=0:0.1:14;

%isoelectric point calculation
pI=0;
k=2;
while pI==0
    if Z(k)<=0 && Z(k-1)>=0
        pI = p(k-1) - (Z(k-1)/((Z(k)-Z(k-1))/0.1));
    else
        pI = 0;
    end
    k=k+1;
end

%Hydrophilicity Calculation

Avg_hydrophilicity = mean(Peptide_hydrophilicity);



ZperMass = Z/(Total_MW/1000);

disp(' ')
disp([name,' (',num2str(AA_Count),' Residues)'])
disp(['MW = ',num2str(Total_MW),' Da'])
disp(['pI = ',num2str(pI)])
disp(['Average Hydrophilicity = ',num2str(Avg_hydrophilicity)])
disp(['At pH=3, Z/kDa = ',num2str(ZperMass(31))])
disp(['At pH=4, Z/kDa = ',num2str(ZperMass(41))])
disp(['At pH=5, Z/kDa = ',num2str(ZperMass(51))])
disp(['At pH=6, Z/kDa = ',num2str(ZperMass(61))])
disp(['At pH=7, Z/kDa = ',num2str(ZperMass(71))])
disp(' ')

hold on
```

```
px1=[0 14];
px2=[0 0];
%plot(p,ZperMass,'b--',px1,px2)
plot(p,ZperMass,'ko')
xlabel('pH')
ylabel('Charge per kDa')
```
